# Biophysical insights into glucose-dependent transcriptional regulation by PDX1

**DOI:** 10.1016/j.jbc.2022.102623

**Published:** 2022-10-20

**Authors:** Emery T. Usher, Scott A. Showalter

**Affiliations:** 1 Center for Eukaryotic Gene Regulation, Department of Biochemistry and Molecular Biology, The Pennsylvania State University, University Park, Pennsylvania, USA; 2 Department of Chemistry, The Pennsylvania State University, University Park, Pennsylvania, USA

**Keywords:** intrinsically disordered protein, transcription factor, diabetes, posttranslational modification, gene regulation, biophysics, pancreatic and duodenal homeobox 1, Antp, antennapedia, bHLH, basic helix-loop-helix, bZIP, basic leucine zipper, CAT, chloramphenicol acetyltransferase, ChIP, chromatin immunoprecipitation, CK2, casein kinase 2, co-IP, coimmunoprecipitation, Cul3, cullin 3, DBD, DNA-binding domain, ERK1/2, extracellular signal-regulated kinases 1 and 2, GLP-1, glucagon-like peptide 1, Glut1/3, glucose transporter types 1 and 3, GSIS, glucose-stimulated insulin secretion, GSK3, glycogen synthase kinase 3, GWAS, genome-wide associated study, H3, histone H3, H4, histone H4, HD, homeodomain, HDAC, histone deacetylase, HG, high-glucose, HIPK2, homeodomain-interacting protein kinase 2, HMG, high-mobility group, iapp, islet amyloid polypeptide, IDP, intrinsically disordered protein, IDR, intrinsically disordered region, LG, low-glucose, MODY, mature onset diabetes of the young, NLS, nuclear localization signal, *O*-GlcNAc, *O*-linked N-acetylglucosamine, PASK, per-arnt-sim kinase, PBX1, Pre-B-cell leukemia transcription factor 1, PCIF1, PDX1 C terminus-interacting factor, PDX1, pancreatic and duodenal homeobox 1, POZ, Pox virus and Zing finger, pRB, hyperphosphorylated RB, PTM, posttranslational modification, RB, retinoblastoma, SAPK2, stress-activated protein kinase 2, SPOP, speckle-type POZ protein, SUMO, small ubiquitin-like modifier, T2D, type 2 diabetes, TAD, transactivation domain, TALE, transcription activator-like effector, TF, transcription factor, TSS, transcription start site

## Abstract

The pancreatic and duodenal homeobox 1 (PDX1) is a central regulator of glucose-dependent transcription of insulin in pancreatic β cells. PDX1 transcription factor activity is integral to the development and sustained health of the pancreas; accordingly, deciphering the complex network of cellular cues that lead to PDX1 activation or inactivation is an important step toward understanding the etiopathologies of pancreatic diseases and the development of novel therapeutics. Despite nearly 3 decades of research into PDX1 control of *Insulin* expression, the molecular mechanisms that dictate the function of PDX1 in response to glucose are still elusive. The transcriptional activation functions of PDX1 are regulated, in part, by its two intrinsically disordered regions, which pose a barrier to its structural and biophysical characterization. Indeed, many studies of PDX1 interactions, clinical mutations, and posttranslational modifications lack molecular level detail. Emerging methods for the quantitative study of intrinsically disordered regions and refined models for transactivation now enable us to validate and interrogate the biochemical and biophysical features of PDX1 that dictate its function. The goal of this review is to summarize existing PDX1 studies and, further, to generate a comprehensive resource for future studies of transcriptional control *via* PDX1.

## PDX1 biology & background

### PDX1 in development and glucose homeostasis

**Figure 1:**
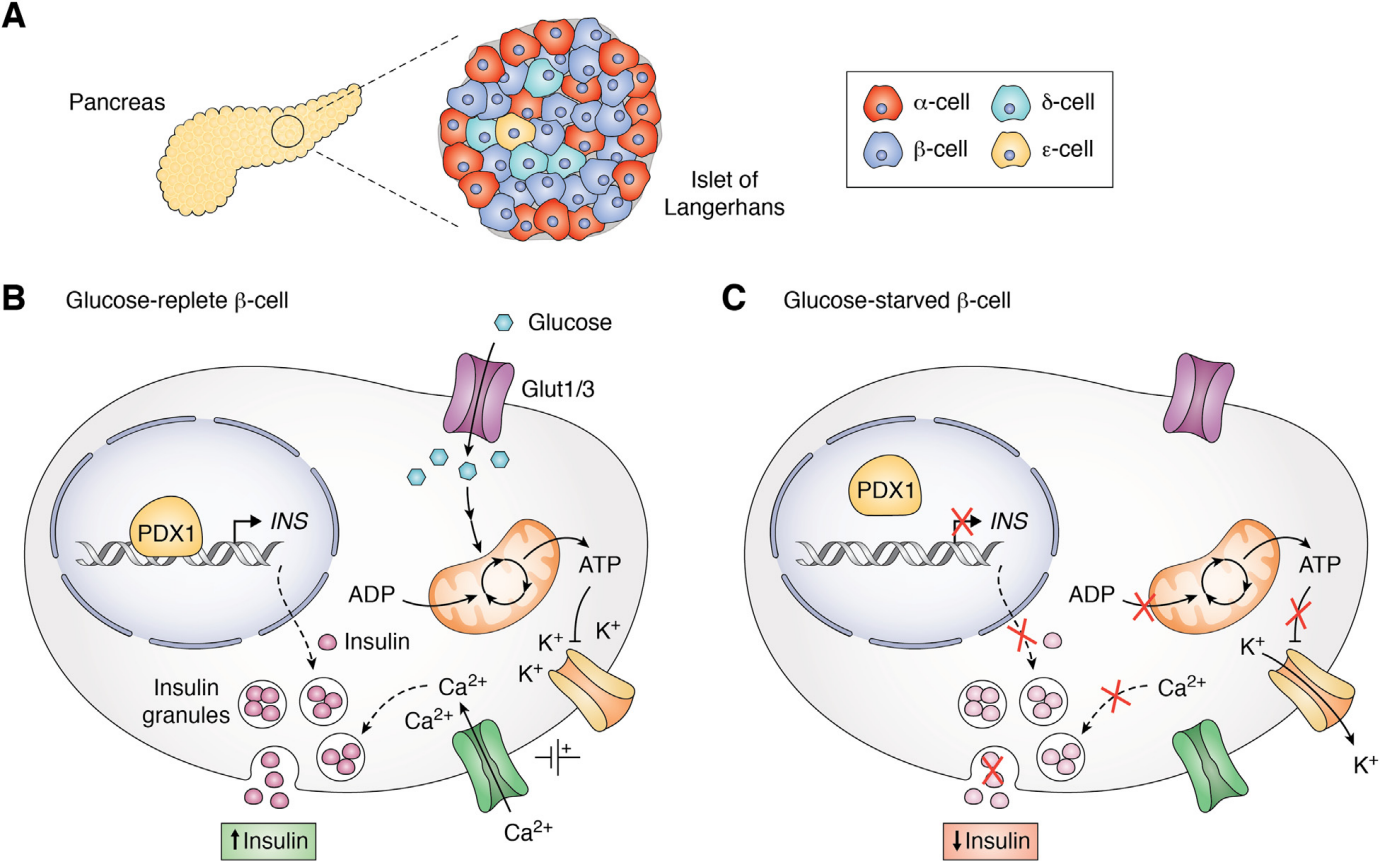
**β cells comprise the majority of islet cell mass in the pancreas.***A*, cartoon representation of the *H. sapiens* pancreas and composition of the islets of Langerhans. *B*, simplified diagram of β cell action in high and *C*, low glucose conditions. Briefly, glucose enters the cell *via* the glucose transporter, Glut1/3 (*magenta*), and enters glycolysis. The pyruvate product is processed by the Krebs cycle in the mitochondria, thereby generating ATP. ATP inhibits the potassium efflux pump (*yellow*), which causes K^+^ ion build-up in the cell. Consequently, membrane polarization permits calcium uptake *via* a voltage-gated ion channel (*green*), which drives exocytosis of insulin granules (*pink*). In tandem, glucose sensing by the β cell stimulates PDX1 (*tan*) DNA binding and INS gene activation.

The pancreatic and duodenal homeobox 1 (PDX1) (also known as GSF (1), IDX-1 (2), IPF1 (3), IUF (4), and STF-1 (5)) is the central activator of glucose-dependent insulin transcription in pancreatic β cells (4). The *PDX1* gene is located at chromosome 13q12.1 in *Homo sapiens* and at 5qG3 in *Mus musculus* (mouse); unless otherwise specified; discussion herein will focus on the *H. sapiens* protein PDX1. In addition to its role in developmentally mature organisms, PDX1 is of particular importance in the early differentiation of gut endoderm cells into pancreatic progenitors and, subsequently, the formation of insulin-producing cell mass (6). Impaired expression or certain mutations of *PDX1* during embryonic development are linked to pancreatic agenesis and the onset of diabetes with age (7, 8, 9, 10). PDX1 is one of several transcription factors that direct development of pancreatic progenitor cells into the α, β, δ, and ε cells of the islets of Langerhans (11) (Fig. 1*A*; for a thorough review about the different islet cell types, we refer the interested reader to reference (12)). Importantly, PDX1 supports phenotypic maintenance of β cells, which enables glucose-stimulated insulin secretion throughout the organism’s lifetime (13, 14). To this end, PDX1 plays a repressive role in β cells by downregulating non-β cell genes (15, 16). In addition, chromatin immunoprecipitation (ChIP) sequencing experiments using different islet cell types identified a strikingly similar PDX1 genomic distribution in β and δ cell (17, 18). In this review, we focus on PDX1 control of *Insulin* transcription but acknowledge its many and diverse regulatory roles in pancreatic health and disease.

In developmentally mature organisms, PDX1 expression is primarily localized to β cells. Notably, even among the β cells in a given islet, PDX1 expression is variable across transcriptionally mature and immature cells. This heterogeneity is important for glucose-stimulated insulin secretion and metabolism by the collective β cell population (19, 20). PDX1 regulates the expression of insulin and other hormones in the pancreas such as somatostatin, glucokinase, glucose transporter type 1/3 (Glut1/3), and islet amyloid polypeptide (21, 22, 23, 24, 25). However, given the importance of insulin in the function of β cells and the abundance of studies to this end, PDX1 control of *INS* transcription will be the focus of this review.

While not the specific focus of this review, glucose sensing and insulin secretion by the β cell are inherently related to PDX1 action; accordingly, we provide a high level summary of these β cell processes. Briefly, Glut1/3 mediates glucose transport into the β cell, after which the glucose is processed by glycolysis in the cytoplasm and through the Krebs cycle in the mitochondria. The resulting increase of cytoplasmic ATP concentration blocks potassium efflux, leading to a change in membrane polarization that ultimately stimulates exocytosis of insulin granules (26, 27) (Fig. 1*B*, top). Increased insulin in response to glucose stimulus is primarily a consequence of translation of proinsulin mRNA, thus replenishing the cell’s insulin stores (28). In contrast to our deep and evolving understanding of glucose-stimulated insulin secretion, the specific mechanisms that lead to PDX1 activation are incomplete. In stimulating glucose conditions, PDX1 and other transcription factors localize to the nucleus and together activate transcription of *INS* mRNA (29, 30, 31, 32). On the other hand, low glucose conditions halt insulin secretion and inhibit PDX1-driven transcriptional activation (33, 34) (Fig. 1*B*, bottom). To gain insight into how PDX1 orchestrates *INS* transcription, we must first interrogate the many reported PDX1 interactions and modifications associated with its activating function.

## PDX1 intrinsically disordered regions orchestrate protein function

### Disorder in transcription factors

In addition to cooperatively folded DNA-binding domains (DBDs), transcription factors tend to contain intrinsically disordered transactivation domains (TADs) that support interaction with posttranslational modification (PTM) enzymes, chromatin remodelers, transcriptional coactivators or repressor proteins, and other transcription factors (35, 36, 37, 38, 39, 40). Intrinsically disordered proteins (IDPs) or intrinsically disordered regions (IDRs) are central regulators of protein function in nearly all cellular processes but are especially enriched in signaling and transcription-associated processes (41, 42). Up to 95% of eukaryotic transcription factors contain IDRs of >30 residues compared to up to 55% in control, nontranscription factor proteins (43). Although IDRs lack stable folded structures, conformational plasticity poises them for interaction with multiple and/or functionally diverse binding partners; consequently, understanding IDR intermolecular interactions in the context of cellular function and health requires evaluating their solution ensembles with high spatial and temporal precision (36, 44, 45). This is the exact paradox—the need to quantitatively describe structure in the context of pervasive disorder—that has impeded molecular characterization of transactivation function of transcription factors.

Many IDR interactions are tailored by their covalent modification in or near a binding surface; such modification may be coupled to conformational consequences, but this is not universally required (46, 47). Due to their solvent accessibility and relative enrichment in modifiable amino acids, IDRs are frequently the subjects of PTMs in the pursuit of further functional regulation (48). As will be discussed in the following sections of this review, several PDX1 interactions are maintained through IDRs and PDX1 PTMs tend to cluster in or near known interaction sites (see Table 1, Table 3). As such, the unstructured regions of PDX1 are valuable targets of study toward understanding basic mechanism and potential therapeutic strategies.

**Table 1:** Proteins reported to directly associate with PDX1 for the regulation of glucose-stimulated insulin transcription and their roles in INS regulation

Factor	Ontology	Reported function with PDX1	PDX1 AAs for interaction	Condition	Ref.
β2^a^	bHLH TF	Binds E-box motifs^a^ & Pdx1 *via* bHLH domain	138–213 (HD)	HG	(95, 195)
Brg1^b^:Swi/Snf	ATPase	Remodels chromatin for promoter accessibility	*NS*	HG	(75)
Bridge-1^e^	PDZ-domain coactivator	Binds Pdx1 and E2A to enhance *INS* activation	1–63	HG	(100, 101)
Brm^b^:Swi/Snf	ATPase	Represses *INS* activation by heterochromatin formation	*NS*	LG	(75)
CBP^d^	Acetyltransferase	Leads to histone acetylation	13–73 (TAD)	HG	(196)
E47^a^	bHLH TF	binds E-box motifs^a^ & Pdx1 *via* bHLH domain	138–213 (HD)	HG	(95, 195)
HDAC-1/-2	Deacetylase	Deacetylates H4 to induce chromatin compaction (part of NurD complex)	80–283 (post-TAD)	LG	(31, 75, 112)
HMGA1	AT-hook TF	Enhances *INS* activation by Pdx1	138–213 (HD)	HG^c^	(95)
Importin β1	Importin	Facilitates Pdx1 nuclear import	146–205 (HD)	HG	(66)
JMJD3	Demethylase	Removes repressive H3K27Me3	*NS*	HG	(83)
MafA	bZIP TF	Binds C1 elements & augments *INS* transcription	*NS*	HG	(99)
p300^a^^,^^d^	Acetyltransferase	Leads to H4 hyperacetylation	13–22, 32–38, 60–73 (TAD)	HG	(87, 88)
RB	tumor suppressor	stabilizes & prevents Pdx1 ubiquitination	153–167 (in HD)	HG	(70)
Set7/9	Methyltransferase	Activates *INSs* via H3 & Pdx1 methylation	1–205 (N-term & HD)	HG	(39, 82)
Sox6	HMG TF	Represses Pdx1-driven GSIS	1–144 (N-term)	LG^c^	(115)
SPOP	Ubiquitin ligase adapter	Mediates proteasomal degradation	224–236 & 265–275 (C-term)	LG	(34, 118)

bHLH, basic helix-loop helix DNA-binding domain; bZIP, basic leucine zipper domain; GSIS, glucose-stimulated insulin secretion; HG, high glucose; LG, low glucose; NS, not specified; TALE, transcription activator-like effector; TF, transcription factor.

a E47 (aka E2A) and β2 (aka NeuroD1) form a heterodimer (often called E47/β2) to bind E-box elements; E47/β2 also interacts with p300.

b Brg1 and Brm incorporation into Swi/Snf is mutually exclusive.

c The study did not investigate the glucose conditions that give rise to this interaction, but glucose conditions were inferred from context.

d p300 and CBP have high sequence homology and are often interchangeably involved in gene activation.

e Bridge-1 was cloned from the INS-1 (rat insulinoma) cell line; PSMD9 is the human homolog.

### A cooperatively folded DBD amid disorder

**Figure 2:**
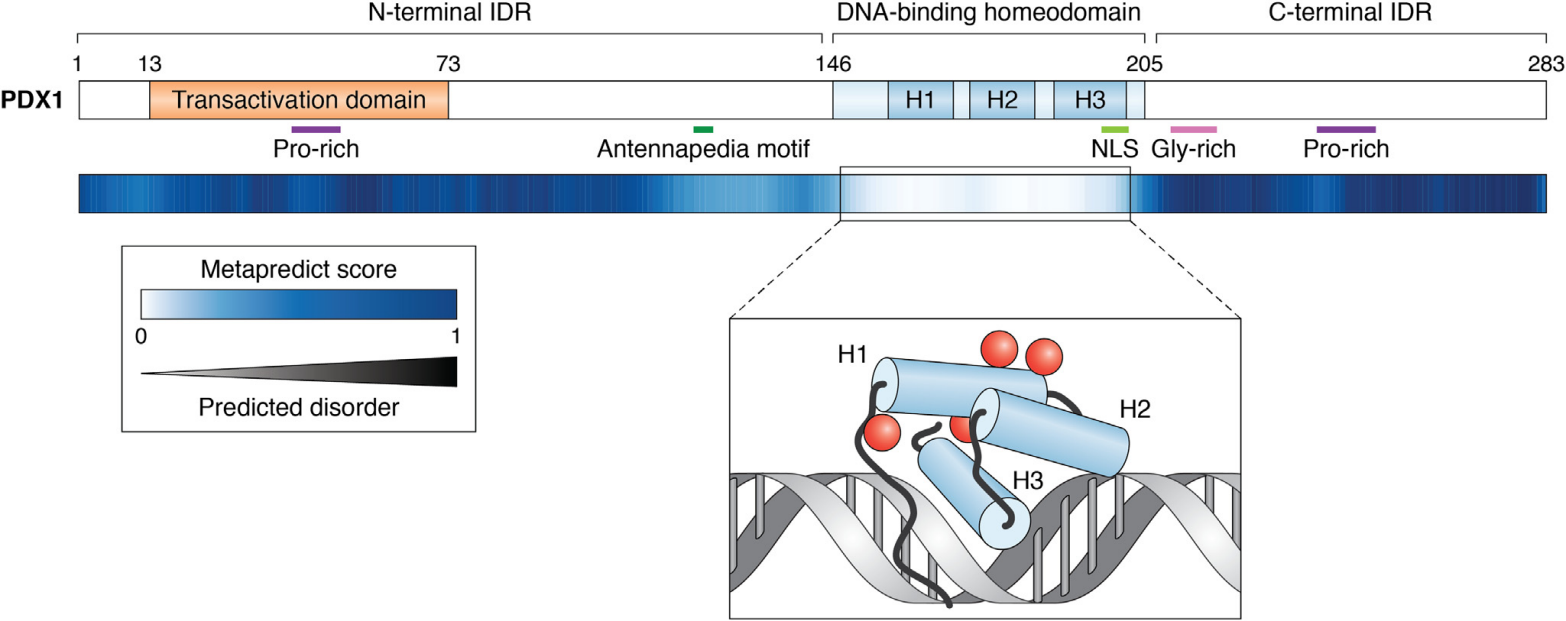
**Excepting the DNA-binding homeodomain, PDX1 is primarily intrinsically disordered.** Each residue of PDX1 is colored by the predicted degree of disorder such that 0 (*white*) is highly ordered and 1 (*dark blue*) is highly disordered (58). Notable features are annotated with colored bars: the transactivation domain (TAD) is shown in *orange* (residues 13–73). An Antennapedia (Antp) hexapeptide motif and nuclear localization signal (NLS) are shown in *green*. Regions of sequence bias in the N- and C-terminal intrinsically disordered regions (IDRs) are shown in *purple* (Pro-rich) and *pink* (Gly-rich). The inset panel depicts a diagram of the DNA-binding domain (adapted from PDB ID: 2H1K (49)). The three α helices are labeled H1, H2, and H3, from N to C terminus, respectively. *Red spheres* indicate the locations of residues in Helix 1 that comprise the RB interaction motif (70). PDB, Protein Data Bank; RB, retinoblastoma.

The human PDX1 protein consists of 283 amino acids and three structurally distinct domains (Fig. 2). The DNA-binding homeodomain (HD) shares remarkable sequence and structural homology with other Hox and ParaHox proteins that also bind DNA (49, 50). Amino acid deviation from the consensus HD sequence grants sequence specificity for DNA binding. The PDX1 HD binds the core DNA motif 5′-CTAATGAG-3′, which is found upstream of the numerous genes for which PDX1 controls expression (51). Different HD-containing proteins derive DNA-binding specificity, in part, by the sequences of nucleotides flanking the TAAT motif. Indeed, sequence variation across *Insulin*, *iapp*, and *glucokinase* gene promoter elements permits differential PDX1 binding to the various promoter sites (51, 52, 53). The PDX1-HD comprises only 60 of the 283 residues in the full-length protein; the remaining two domains of the protein, the N- and C-terminal domains, are primarily intrinsically disordered and sustain the protein–protein interactions that fine-tune PDX1 functions.

### Regulation of PDX1 function *via* IDRs

Amino acids 1 to 145 comprise the N-terminal intrinsically disordered domain of PDX1 (PDX1-N) (Fig. 2). Amino acids 13 to 73 are annotated as a transactivation domain based on the transactivation ability of PDX1 deletion mutants and the recruitment of coactivating factors to this region (54, 55, 56). An *in vitro* study of the full-length human PDX1 protein by CD spectroscopy suggested a random coil content of almost 50%. The remaining populations were about equal fractions α-helix, β-sheets, and turns (57). Although the specific conformational properties of PDX1-N have not yet been described experimentally, amino acid bias in this region and computational disorder prediction tools suggest that this region is not globular, which is consistent with the calculated secondary structure populations determined by CD experiments on the full-length protein (57).

The disorder prediction from the ‘metapredict’ online tool is plotted per residue in Fig. 2, wherein white residues have low predicted disorder and black residues have high predicted disorder (58). Of the sequence space found in PDX1 residues 1 to 145, prolines represent ∼20%, alanines comprise ∼14%, and glycine and glutamate each represent about 8% (UniProt #P52945). Proline, glycine, and charged and polar amino acids are generally considered ‘disorder promoting’; in contrast, hydrophobic residues tend to be associated with folded protein structures (59). Alanine, although it contains a methyl group side chain, is overrepresented in IDPs compared to folded proteins and does not necessarily fall under either ‘disorder-promoting’ or ‘fold-promoting’ (60, 61).

The C terminus of PDX1 (PDX1-C, residues 205–283) is also intrinsically disordered; in contrast to many IDPs and IDRs, which have some degree of transient or low population secondary structure, solution-state studies of PDX1-C by NMR spectroscopy and small angle X-ray scattering support the disorder prediction that this region is devoid of persistent structure (Fig. 2) (62). PDX1-C is rich in glycine and proline residues, which make up about 15% and 22% of the sequence, respectively, and contribute to its IDP behavior.

In contrast to the diverse PDX1-N interactome (see the *protein–protein interactions* section), PDX1-C has only one annotated binding partner, speckle-type POZ protein (SPOP) (also known as PCIF1), which facilitates PDX1 proteasomal degradation (34, 63). Although downregulation of PDX1 *via* its C-terminal IDR implicates this region as an inhibitory domain, PDX1-C may play a secondary activating role toward the expression of specific PDX1-controlled genes, as deletion of the C-terminal domain impaired transactivation ability by PDX1 (64). Early studies of the transactivation potential of PDX1 deletion constructs suggest that PDX1-C may contain yet unidentified transactivation domains; this hypothesis is supported by the high sequence conservation and disease-associated mutation prevalence in PDX1-C (64, 65).

## Protein–protein interactions

PDX1 binds to A-box and other promoter elements by its canonical helix-turn-helix DNA-binding HD (49, 51). In addition to DNA, the PDX1 HD reportedly binds to other protein factors that serve to control PDX1 function. Notably, however, many PDX1 protein–protein interactions are maintained by its two intrinsically disordered domains, which are discussed later. Of particular, importance to gene activation by PDX1 is the N-terminal IDR. The PDX1 N-terminal TAD has been functionally annotated as a necessary site of assembly for several of the factors that work in synergy with PDX1 toward *INS* activation, whereas the C-terminal IDR serves as an interface toward PDX1 degradation (34, 54).

In response to glucose stimulus, the PDX1 HD is engaged by the nuclear import receptor, importin β, to facilitate PDX1 nuclear localization and subsequent activation (66, 67). The PDX1–importin interaction was demonstrated by pull-down experiments using *in vitro* translated PDX1 and GST-tagged importin β. In cells, disruption of the importin β nuclear transport axis led to PDX1 accumulation at the nuclear pore complex and, consequently, impaired transactivation by PDX1 (66). Although the specific signals that lead to PDX1 binding (and unbinding) by importin β are not well established, kinase inhibition studies suggest that phosphorylation events in the PDX1 HD and C-terminal IDR may influence subcellular localization. In this vein, PDX1 translocation may be prompted by glucose-linked phosphorylation or dephosphorylation events that present or occlude an importin-binding site on PDX1 (66, 68, 69).

Separately, the Retinoblastoma (RB) protein, which is a well-known tumor and cell cycle suppressor, interacts with a specific structural motif within PDX1-HD. The PDX1-RB interaction was identified by overexpression in and coimmunoprecipitation (co-IP), although the experiments were performed in a non-β cell line. Hyperphosphorylated RB (pRB) associates with the Pdx1 RB interaction motif found in Helix 2 of the HD fold (see Fig. 2) and does so preferentially in high glucose conditions. Mutation of the RB interaction motif domain destabilizes PDX1 by promoting its proteasomal degradation, suggesting that pRB binding to PDX1 confers stability and increases PDX1 half-life (70).

There is little available information describing specific molecular mechanisms for PDX1 association with its various interaction partners. Thus, it is imperative that efforts be directed to understanding the atomistic determinants of binding and the cellular signals (such as glucose stimulus) that promote or preclude interactions with PDX1. A major barrier to understanding the regulatory roles of PDX1-interacting factors is the absence of high-resolution structural information describing the binding interfaces. The X-ray crystal structure of the PDX1 HD in complex with DNA led to a model for induced fit for PDX1 recognition of and binding to DNA elements (49). Crystal structures of peptides derived from the PDX-1 C-terminal IDR in complex with the negative regulator SPOP showed that this domain of PDX1 remains extended upon binding to the substrate-binding domain of SPOP (71, 72). At the time of writing, there are no structures of PDX1—alone or in complex—to describe the numerous interactions maintained by the PDX1 N-terminal IDR. The remainder of this section reviews selected PDX1 interaction partners in need of structural characterization to validate existing evidence for interaction and describe specific mechanisms of binding.

### PDX1 activation

Given stimulating glucose, the PDX1 N-terminal IDR recruits chromatin remodeling enzymes to facilitate promoter accessibility. In addition, several coactivating transcription factors (described later) associate directly or indirectly with PDX1; some such factors bind nearby promoter or enhancer elements toward activation of *INS* transcription (29, 73).

The chromatin remodeling complex Swi/Snf (termed “Brg1- or Brm-associated factors” or BAF, in humans) alters nucleosome positioning by sliding or ejecting nucleosomes to facilitate transcriptional activation or repression (74). The interaction between PDX1 and multiple subunits of Swi/Snf within a mouse insulinoma cell line was identified through mass spectrometry of complexes crosslinked with PDX1 (75). During development or given glucose-replete conditions in β cells, the complex comprising ATPase subunit Brg1 associates with PDX1. Notably, mutually exclusive incorporation of Brg1 *versus* Brm occurs at genes (including *INS*) that are activated by PDX1 (38, 75). In the context of human insulin-positive β cells isolated from type 2 diabetes patients, proximity ligation of PDX1 with Brg1:Swi/Snf showed that their association is impaired compared to cells isolated from healthy patients (75).

Although it is clear Swi/Snf is required for adequate insulin production, the specific glucose-driven signals that lead to Brg1:Swi/Snf association with PDX1—and the physical basis for this interaction—are not experimentally established (76). The Swi/Snf subunit SMARCB1 (also known as BAF47, INI1, or SNF5) was prominent among the subunits identified from crosslinking mass spectrometry experiments with PDX1. In addition to its assembly with the other subunits of Swi/Snf, SMARCB1 interacts *in vitro* and *in vivo* with the proline-rich domain of an Epstein-Barr viral protein, EBNA2 (77). Further, EBNA2 disrupts the interaction between SMARCB1 and a proline-rich repetitive region of apoptotic factor GADD34 (78). Given these examples, we might envision interaction of SMARCB1 with one of the two proline-rich IDRs of PDX1. SMARCB1 also interacts with the transcription factor MYC within the MYC/MAX heterodimer but interaction with MYC precludes its DNA binding (79); importantly, PDX1 retains DNA-binding ability when in complex with Swi/Snf (75).

PDX1 also recruits PTM enzymes that act upon histones H3 and H4 toward chromatin opening under stimulating glucose conditions. One such enzyme is the methyltransferase Set7, which associates with PDX1 *via* the N-terminal TAD and installs an ‘activating’ methyl group on Lys4 of H3 (H3K4). In contrast to the established role of Set7 as a monomethyltransferase, dimethylated H3K4 was the primary species identified at active *INS* promoters, which implies the involvement of a yet unidentified secondary methyltransferase enzyme (80, 81). As discussed further, PDX1 is one of many reported methylation substrates of Set7 and, as such, it is reasonable to hypothesize that the interaction between PDX1 and Set7 involves the N-terminal IDR of PDX1 containing the modified lysine residues (Lys123 and Lys131) (82).

High glucose conditions also reportedly drive the association of PDX1 with the demethylase, JMJD3, toward removal of ‘repressive’ methyl groups from H3K27, but it is unclear which domain of PDX1 maintains this interaction (83). In the case of JMJD3 binding to p53, the interaction is mediated, at least in part, by the p53 C-terminal tetramerization domain (84). In addition, the role of JMJD3 in this context may involve demethylation of p53 itself (85). Importantly, structural studies of PDX1 show no evidence for structured regions analogous to the p53 tetramerization domain, nor are there reports for direct action of JMJD3 on PDX1. The HD-containing transcriptional activator ISL-1 is reported to act alongside PDX1 at *INS* enhancers; co-IP experiments showed an interaction between ISL-1 and JMJD3 that requires the ISL-1 HD (86). It is possible that a similar interaction between JMJD3 and the PDX1 HD may contribute to the recruitment of JMJD3. Importantly, the existing data do not rule out a model in which crosstalk between PDX1 and ISL-1 contribute to JMJD3 action on repressive histone modifications.

The PDX1 TAD also recruits the acetyltransferase p300 to PDX1-activated promoters. Specifically, p300-driven transcriptional activation was markedly improved through hyperacetylation of histone H4, supported by cooperative assembly of PDX1 and the transcription factors E47 and β2 (discussed further) (87, 88). Like nearly all PDX1 complexes, the PDX1-p300 interaction is glucose dependent; however, p300 association with PDX1 is additionally regulated by the glucose-dependent phosphorylation state of the PDX1 TAD. Accordingly, phosphatase inhibition in β-like cells led to the constitutive interaction of PDX1 and p300 (88). Mice lacking p300 (and its homolog CBP) had reduced β cell mass from impaired proliferation in development and were glucose intolerant arising from lowered insulin secretion. In this case, the phenotypes were primarily associated with the loss of the ‘activating’ H3K27-acetyl modification (88). Further, siRNA knockdown of PDX1 in an insulinoma cell line led to reduced promoter occupancy of p300. In the same study, ChIP quantitative RT-PCR of RNA Pol II showed that promoter occupancy was not affected by PDX1 knockdown, whereas occupancy within the gene body was diminished (89).

**Figure 3:**
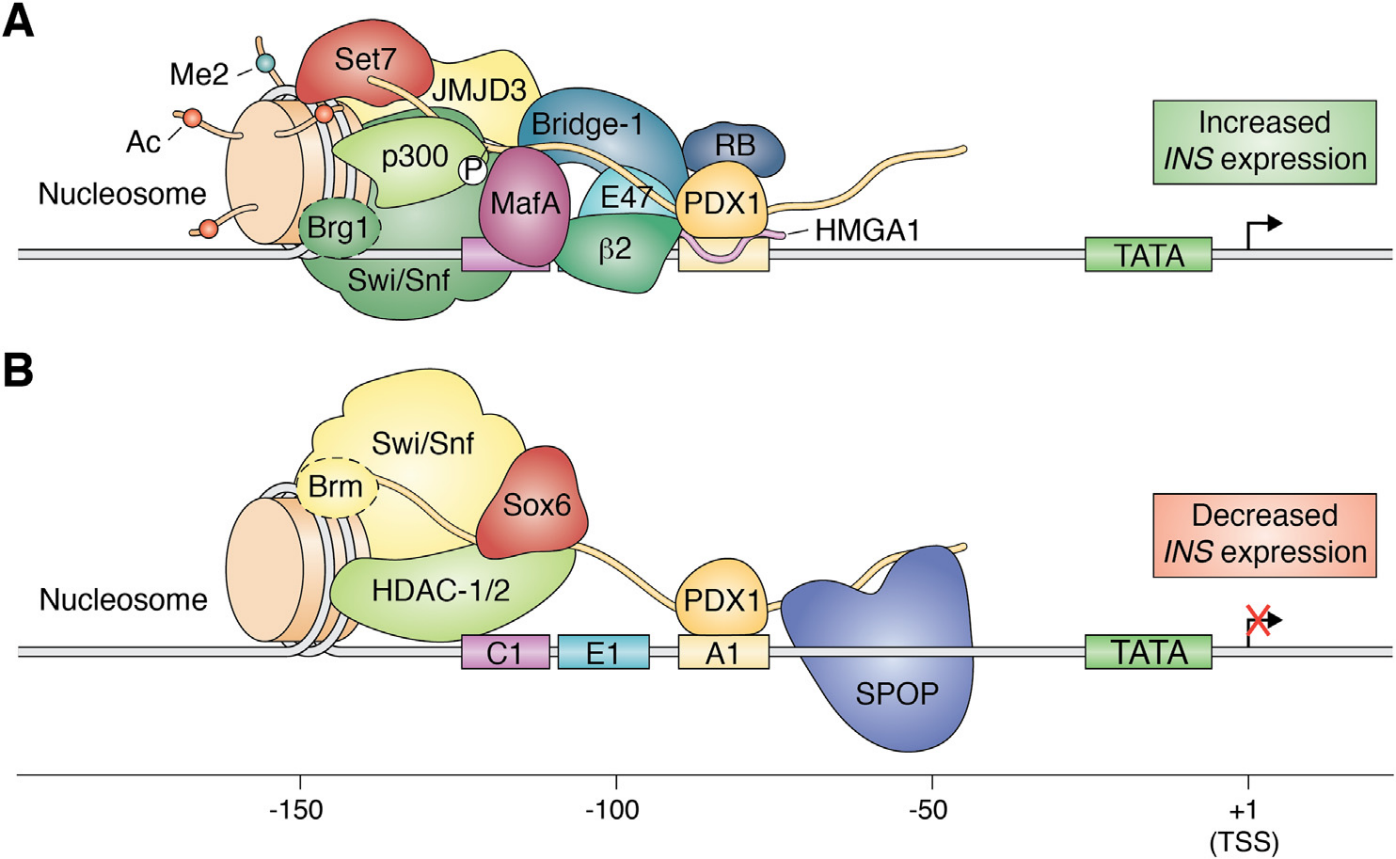
**Summary diagrams of protein complexes formed at the human insulin promoter.** (*A*) Pdx1-interacting factors associated with stimulating glucose conditions. (*B*) Pdx1-interacting factors associated with depleted glucose conditions. Note that the interacting factors are assembled based on the assignment for their interaction with one of the PDX1 domains; for all proteins except RB and SPOP, the specific interaction footprint is not yet known. Ac, acetyllysine; Me2, dimethyllysine; RB, retinoblastoma; SPOP, speckle-type POZ protein; TSS, transcription start site.

Taken together, we may consider a model for p300 coactivation of PDX1-controlled genes upon p300 recruitment by the phosphorylated Pdx1 TAD (Fig. 3*A*). Informed by structural studies of nonhistone proteins in complex with p300, we hypothesize that PDX1 binding occurs in a similar fashion. In the case of E47, two small helical domains of p300, TAZ1, and TAZ2 compete for binding to an acidic IDR of E47 (90). p300 interacts with acidic, leucine-rich IDRs of several transcription factors *via* its TAZ or KIX domains; this interaction is often coupled to a disorder-to-order transition upon binding. Additionally, although there is poor sequence conservation across the different p300 binding partners, these motifs are generally rich in leucine or other hydrophobic residues (91). As such, we might expect that the PDX1 N-terminal IDR harbors a motif that satisfies the flexible binding motif for a p300 TAZ domain. Of additional importance, phosphorylation near TAZ domain-interacting motifs of other transcription factors, including p53 and CREB, enhances binding by p300 and subsequent transactivating potential (92, 93). Thus, it is reasonable to predict that p300 associates with the leucine-containing region of the PDX1 N-terminal IDR that is adjacent to phosphorylated serines 61 and 66. Contradictory to the latter hypothesis, deletion of a glutamine- and proline-rich domain in the C-terminal region of p300 abrogated binding to *in vitro–*translated PDX1 (87). Although this result may reflect a non-native complex between p300 given an unphosphorylated form of PDX1, we do not exclude the possibility that the PDX1 TAD may associate with p300 in a manner distinct from that of other transcription factors.

Among the PDX1 coactivating factors are a number of other DNA-associated transcription factors that, in concert, enable robust *INS* activation by Pdx1. Two such factors are E47 (also called Pan1) and β2 (also called NeuroD1); the E47/β2 heterodimer binds E-box motifs near to Pdx1-bound A-box DNA motifs (5, 94). Each E47 and β2 reportedly interact directly with PDX1 *via* an HD-containing region (residues 138–213) based on pull-down experiments with *in vitro*–translated PDX1 constructs (95). Interestingly, deletion of the PDX1 N-terminal TAD did not impact binding to E47, but it is possible that recombinant or *in vitro*–translated PDX1 lacked glucose-linked phosphorylation marks within the TAD that could contribute to E47/β2 binding. Indeed, phosphorylation within the TAD of transcription factor MafA enhanced its interaction with β2 in cells (96). PDX1, E47, and β2 each also directly interact with p300 *via* their respective TADs, which support a model through which the PDX1-E47/β2 complex cooperatively recruits p300 or other chromatin remodelers to the *INS* promoter (87, 97).

The β cell–specific transcription factor, MafA, which binds the C1 element of the *INS* promoter, is integral to *INS* activation alongside PDX1 (29, 98). Notably, co-IP experiments showed that MafA physically associates with PDX1 and β2 but not p300. The PDX1 domain involved in interaction with MafA was not determined (99). The same study reported cooperativity between MafA, PDX1, and E47/β2 toward *INS* activation in rat and mouse insulinoma cell lines.

In contrast to the DNA-associated factors described previously, the PDX1 TAD also associates with a coactivator protein, Bridge-1 (the human homolog of Bridge-1 is proteasomal subunit PSMD9). Although Bridge-1 does not appear to have transcription factor activity, transcription reporter assays and pull-down studies showed that it associates with both E47 (but not β2) and PDX1 toward enhanced *INS* activation (100, 101). To our knowledge, Bridge-1 has not been described elsewhere in a coactivating role. In contrast, overexpression of Bridge-1 in transgenic mice was detrimental to *INS* transcription, β cell death, and broadly disrupted postnatal pancreatic health and tissue morphology (102). A proteomic study in human islets in found that Bridge-1/PSMD9 was among the proteins with modestly increased expression in response to insulin treatment. The same study noted that, in line with the annotation of PSMD9 as a regulatory subunit of the proteasome, its expression was primarily localized to the cytosol (103). Taken together, it is possible that Bridge-1/PSMD9 stimulatory action toward PDX1 is determined by the context- and compartment-dependent expression of Bridge-1. Further, we should not exclude a model in which aberrant overexpression of Bridge-1/PSMD9 is involved in the PDX1 proteasomal degradation pathway or is otherwise implicated in β cell mass loss and diabetes.

The high mobility group A1 protein (HMGA1, also known as HMG-I/Y) is an architectural factor that binds to DNA and other transcription factors but does not itself have intrinsic transcription factor activity. Instead, HMGA1 binds AT-rich minor grooves and modulates the DNA conformation toward additional transcription factor recruitment and binding (104, 105). HMGA1 is primarily intrinsically disordered, and its DNA-binding ability is related to phosphorylation-dependent changes to its conformational ensemble (106, 107). In addition, HMGA1 is a negative regulator of the p53 protein family through direct interaction with the p53 C-terminal tetramerization domain (108, 109).

Loss of *HMGA1* impairs endocrine pancreas function and is associated with the development of insulin resistance and diabetes. Indeed, *HMGA1*-deficient patients exhibited impaired insulin secretion and reduced binding to the *INS* promoter by PDX1 (110). ChIP quantitative RT-PCR experiments in a rat insulinoma cell line demonstrated HMGA1 occupancy at the *INS* promoter and pull-down experiments using *in vitro*–translated PDX1 showed association between HMGA1 and PDX1 (110, 111). Alongside another factor, MafA, overexpression of HMGA1 improved the glucose-dependent transactivation potential of PDX1 through HMGA1 interaction with the *INS* promoter and directly with the PDX1 HD (95, 111) (Table 1). Although HMGA1 interaction with p53 has been established in cells and *in vitro*, it is not clear that the binding mode toward PDX1 will be analogous. However, in the case of p53, interaction of the tetramerization domain with triply phosphorylated HGMA1 involves charge complementarity *via* a basic region of p53 (108). Thus, we propose HMGA1 may leverage the basic stretch of residues directly C-terminal to the PDX1 HD toward complex formation.

While it is clear that there are several protein networks involved in activation of PDX1 transcription factor activity, we must continue to interrogate the binary and higher order complexes that PDX1 forms and link them to specific cellular signals. In doing so, we may establish roles for PDX1 binding partners in healthy and disease contexts. Based on the information available at the time of writing, a possible model for PDX1 assembly with its coactivating factors on the *INS* promoter, in which the PDX1 N-terminal IDR mediates assembly of protein factors for transcription activation, is shown in Fig. 3*A*.

### PDX1 downregulation

In glucose-starved conditions, expression of *insulin* is downregulated: PDX1 recruits chromatin remodeling enzymes to promote chromatin compaction and subsequent *INS* repression near the promoter site (75, 112). As discussed previously, the protein interactions that lead to PDX1 activation of *insulin* are tailored by glucose levels in the β cell. Conversely, in conditions of low glucose, the PDX1 transcriptional complex is dissolved, and these factors are replaced by corepressor proteins (modeled in Fig. 3*B*). In contrast to the several factors that are involved in *INS* activation *via* PDX1, there are fewer known proteins that serve as corepressors.

Similar to recruitment of chromatin remodelers by PDX1 to drive euchromatin formation, PDX1 also recruits remodeling enzymes that promote heterochromatin formation. Histone deacetylases HDAC-1 and HDAC-2 (grouped herein as “HDAC-1/2”), which generally are contained within larger complexes, such as NuRD, remove histone acetylation marks at repressed genes (113). Indeed, in conditions of low glucose, acetylated-H4 and p300 occupancy at the *INS* promoter decrease alongside a concomitant increase in occupancy by both HDAC-1 and HDAC-2 (112).

Perhaps expectedly, given the phosphorylation-dependent recruitment of an acetyltransferase, HDAC-1/2 binding to PDX1 relies on TAD dephosphorylation. Although the phosphatase(s) responsible for PDX1 dephosphorylation have not been experimentally validated, treatment of cells with an inhibitor of the phosphatases PP1 and PP2A disrupted HDAC-1/2 interaction with PDX1. Notably, however, deletion of the TAD region leads to constitutive—irrespective of glucose—interaction of PDX1 with HDAC-1/2 (112). In other words, lacking the TAD containing a phosphorylation switch, HDAC-1/2 associates with PDX1 but does so using residues C-terminal to residue 79; the specific residues on PDX1 involved in the interaction with HDAC-1/2 are not yet known (112). Taken together, we envision a bivalent interaction between HDAC-1/2 and PDX1 in which the PDX1 TAD phosphorylation state and a secondary unidentified interface with PDX1 both contribute to a multifaceted repressive strategy.

The Swi/Snf complex is also recruited to PDX1 under low glucose conditions but now containing the Brm ATPase instead of Brg1 (75, 76). Brm:Swi/Snf and HDAC1 may be recruited together to facilitate gene repression during which Brm:Swi/Snf directs nucleosome positioning (76, 114). Future studies of PDX1 in corepressive roles should emphasize the interaction interface and which residues of PDX1 are engaged with each factor.

Unlike the numerous studies describing PDX1 coactivators, only one corepressor of *INS*, Sox6, has been identified and characterized (115). Sox6 is transcription factor that contains a high-mobility group (HMG) DBD but does not appear to affect *INS* expression through its DNA-binding ability (115, 116). Pull-down experiments *in vitro* showed that the HMG domain of Sox6 associates robustly with PDX1 constructs lacking the C-terminal IDR and does not affect PDX1 DNA binding. Sox6 also bound weakly to the PDX1 N-terminal IDR, but it is not clear whether this region of PDX1 alone is sufficient to sustain an *in vivo* interaction (115). Given these results, it is possible that Sox6 competes for interaction with the PDX1-N with one or more PDX1 coactivators. In a separate study, interaction of the Sox6 HMG domain with the N terminus of HDAC-1 was demonstrated by pull down *in vitro* and by co-IP from non-β cells, suggesting that Sox6 repressive activity could alternatively arise from recruitment of histone deacetylases to PDX1 (117). Although the extent of interplay between repressive PDX1 partners is not clear, there is substantial evidence for the involvement of the PDX1 N-terminal IDR in context-dependent activating and repressive roles; PTM of the N-terminal IDR thus presents a tractable model for such role-switching behavior.

The C-terminal IDR of PDX1 has only one known binding partner—the SPOP. SPOP is a ubiquitin ligase adapter for dozens of IDP substrates and is integral to the proteasomal degradation of PDX1 as a means of repressing transcription factor function (34, 63, 118). Recent studies from our lab and others revealed that SPOP binds two distinct motifs within PDX1-C (71, 72). Within the modular Cul3–ubiquitin ligase complex, SPOP recognizes and binds to protein substrates to promote their ubiquitin-mediated degradation (119, 120). Glucose-dependent association of SPOP with PDX1-C leads to PDX1 turnover in conditions of low glucose (118). Like many IDR interactions, including many of those maintained by PDX1-N, the PDX1 C-terminal domain intermolecular interactions are influenced by PTMs to this region (see section on PDX1 modifications later). Notably, studies of PDX1 and other SPOP-controlled substrates find that phosphorylation in the SPOP-binding motif is refractory to SPOP interaction, but this contrasts with the proposed destabilizing roles for phosphorylation in PDX1-C (71, 121, 122, 123).

To our knowledge, there are no reports of crosstalk between the N- and C-terminal repressive mechanisms that downregulate *INS* transcription. It is similarly not clear whether SPOP-PDX1 interactions occur while PDX1 is still bound to DNA. Notably, PDX1 is the first substrate of SPOP demonstrated to interact in cells in the absence of liquid–liquid phase separation, but it is unknown whether this behavior is a consequence of PDX1 being chromatin bound (72). From the studies of PDX1 downregulation to date, we present a possible model for repression of PDX1 through protein interactions through its disordered termini (Fig. 3*B*).

## PDX1 in disease

### PDX1-associated cancers

PDX1 is implicated in several cancers, although it is not uniformly a tumor suppressor or an oncoprotein (124, 125). In addition to the pancreas, PDX1 is expressed in other cell types to varying degrees. In some cases, such as in the case of the distal stomach, PDX1 is expressed in healthy but not cancerous cells, suggesting its role as a tumor suppressor. Indeed, ectopic expression of PDX1 inhibits cell proliferation in gastric cancer and specific stages of Kras-driven pancreatic ductal adenocarcinoma (126, 127, 128). In the pancreatic progenitor subtype of pancreatic ductal adenocarcinoma, however, PDX1 is expressed in precursor lesions that precede tumorigenesis and metastasis (129, 130, 131). In addition, cBioPortal (https://www.cbioportal.org/) reports PDX1 mutations identified in patients with other cancers, including lung, bladder, uterine, liver, and colorectal cancers (132, 133).

Insulinomas are a class of pancreatic tumors characterized by oversecretion of insulin and, consequently, hypoglycemia. Once thought to be related to PDX1 overactivation of the *INS* gene, DNA methylome analysis of insulinomas *versus* healthy β cells revealed hypermethylation at PDX1-controlled genes, suggesting that *INS* activation in insulinomas uses an alternative promoter and is hijacked by noncanonical transcription factors (134). Notably, these studies do not implicate specific polymorphisms in PDX1 itself, but rather cast PDX1 as a dysregulated contributor among other metastatic driving factors, which underscores the plasticity of PDX1 function tailored by epigenetics and cell-specific conditions.

### PDX1 in diabetes

As a nexus of β-cell response to glucose stimulus, it is unsurprising PDX1 dysfunction, through up/downregulation or mutation(s), is linked to the onset of diabetes (135, 136, 137). Type 1 diabetes is classified as an autoimmune disease, wherein β cell destruction precludes insulin secretion in response to glucose stimulus. Although the resultant hyperglycemia is the same as type 1 diabetes, the underlying contributors to type 2 diabetes (T2D) are elusive (138). Insulin resistance associated with T2D was long believed to arise from poor diet and physical inactivity, but the emergence of genome-wide associated studies (GWAS) enabled the identification hundreds of gene loci correlated with—but not necessarily causative of—predisposition to T2D (139, 140). The models for a role of β cell dysfunction in pathogenesis of T2D are reviewed extensively in reference (141).

Separate from these two well-known forms of diabetes, types of monogenic diabetes account for less than 5% of all diabetes cases and are each linked to defects of a single gene (142). Mutations in PDX1 are associated with T2D and multiple types of monogenic diabetes including Mature Onset Diabetes of the Young (MODY) and neonatal diabetes (143) (Table 2). At the time of writing, 14 types of MODY have been identified; MODY cases associated with mutation(s) to *PDX1* are classified as MODY4 (136, 137, 142, 144). PDX1 is also implicated in pancreatic agenesis, usually arising from *PDX1* mutations that truncate the protein product; PDX1 mutations have even been found in individuals with mutations in other MODY-linked genes (10, 145, 146). At present, however, MODY patients are often misdiagnosed with T2D due to similarities in symptoms and presentation. Future studies of existing and potential novel MODY mutants of PDX1 should focus on establishing causative and/or mechanistic roles for these mutations in order to effectively address therapeutic strategies and treatment plans.

**Table 2:** To-date reported PDX1 mutations found in patients with diabetes

Location in PDX1	PDX1 mutation	Diabetes type	Reported effect/proposed mechanism	Ref.
N-terminal IDR (TAD)	C18R	MODY4, T2D	Reduced *insulin* and *somatostatin* transcripts	(56, 135)
	P33T	MODY4	Reduced *insulin, somatostatin,* & *glucagon* transcripts	(56, 197)
	G55D	MODY4	Not reported	(159)
	Q59L	T2D	Modest reduction in transactivation (CAT reporter)	(136)
N-terminal IDR (post-TAD)	D76N	MODY4, MODY3, T2D	Modest reduction in transactivation (CAT reporter); decreased A3 binding	(135, 136, 158)
	P87L	Neonatal diabetes	Not reported	(152)
	P93R	Neonatal diabetes	Not reported	(152)
	A140T	MODY4	Not reported	(158)
DNA-binding domain	K147R	MODY4	Not reported	(152)
	A152G	Neonatal diabetes	Not reported	(152)
	T154R	MODY4	Not reported	(198)
	R155S	MODY4	Not reported	(153)
	E164D	Neonatal diabetes	Impaired transactivation *via* short Pdx1 half-life	(10)
	F167V	Neonatal diabetes	Not reported	(154)
	R176Q	Neonatal diabetes	Not reported	(152)
	V177M	MODY4	Not reported	(155)
	E178G	Neonatal diabetes	Not reported	(156)
	E178K	Neonatal diabetes	Impaired transactivation *via* short Pdx1 half-life	(10, 157)
	R197H	T2D	Decreased A3 binding & activation	(135)
C-terminal IDR	G212R	MODY4	Reduced transactivation (Gal4 reporter assay)	(145)
	E222K	MODY4	Not reported	(160)
	E224K	MODY4	Reduced transactivation (Gal4 reporter assay); decreased binding to SPOP	(161)
	P239Q	MODY4, MODY3, T2D	Reduced transactivation (Gal4 reporter assay)	(145)
Frameshifts, insertions, and nonsense mutations				
N-term (truncation after 17 residues)	C18stop	Neonatal diabetes	Decreased *insulin* transcription	(152)
157 novel residues; truncation at 191	Ala34fsdelC	Neonatal diabetes	Not reported	(152)
59 novel residues; truncation at 122	Pro63fsdelC	MODY4, T2D, Neonatal diabetes	Localization to cytosol	(65, 144, 146, 149, 150)
C-term (Pro insertion after 243)	InsCCG243	T2D	Modest reduction in transactivation (CAT reporter)	(136)
C-term (G246R), 21 novel residues; truncation at 265	Gly246ArgfsdelC	MODY4	Not reported	(199)

Mutation effects and/or mechanisms for pathogenicity are given for mutations studied *in situ*; mutations detected in diabetics but not linked to phenotype by additional studies are ‘not reported’. Mutations that have predicted, but not validated, effects are given ‘not reported’.

CAT, chloramphenicol acetyltransferase.

### Clinical mutations of PDX1 and their relevance to disease

**Figure 4:**
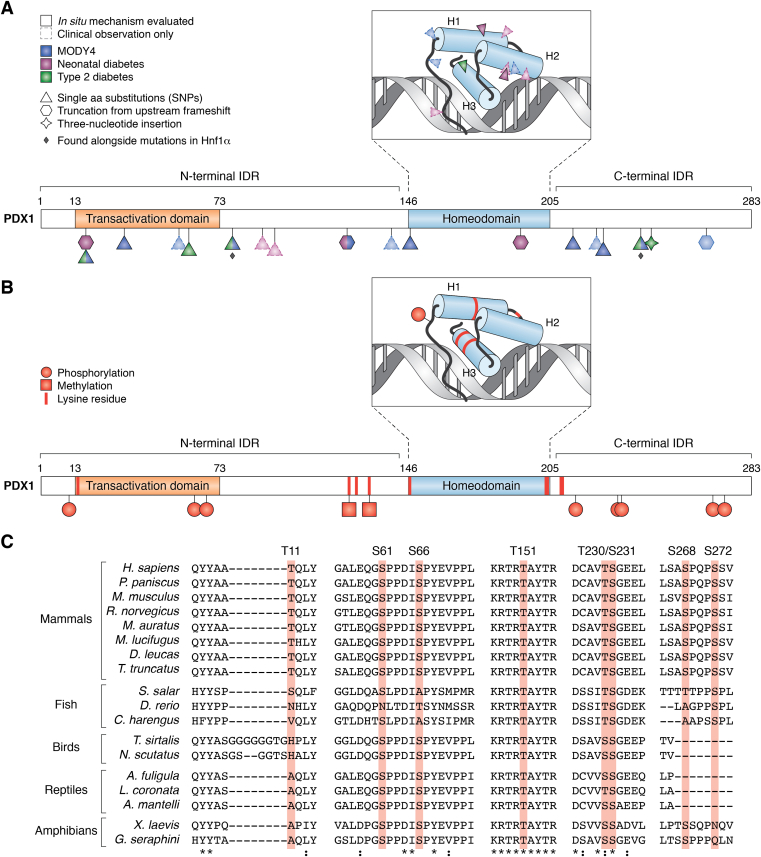
**Schematic representation of the transcription factor PDX1 showing reported clinical mutations and putative posttranslational modifications.** The DNA-binding homeodomain is shown as a 3-D model in the inset of each panel in which H1, H2, and H3 are helices 1, 2, and 3, respectively. *A*, mutation coloring is described in the legend on the left. Mutations with diamonds below their symbols are those found alongside mutations in the MODY3 protein, Hnf1α. Triangles represent the sites of amino acid substitutions arising from single-nucleotide polymorphisms (SNPs). Octagons indicate sites of truncation arising from an upstream frameshift. The star denotes a three-nucleotide insertion resulting in an amino acid insertion (see Table 2). *B*, residues modified by phosphorylation or methylation are denoted with *red* circles or squares, respectively. The locations of lysine residues are indicated by *red* stripes. *C*, multiple sequence alignment of PDX1 from several organisms (mammals, M; fish, F; birds, B; reptiles, R; amphibians, A) using Clustal Omega (194). The regions surrounding each putative phosphorylation site (highlighted in *red* and numbered according to the human PDX1 sequence) are shown. Residues that are 100% conserved are denoted with an asterisk; residues that are conserved with respect to side chain size/chemistry are denoted with a colon. UniProt accession numbers for aligned sequences are P52945, A1YG85, P52946, P52947, P70118, G1PGD4, A0A2Y9N5X8, A0A2U4BGP7, A0A1S3NIB4, F1RBA3, A0A6P3VHE4, A0A6I9WXG3, A0A6J1UKB3, A0A6J3EP98, A0A6J0HJ37, P14837, and A0A6P8RAF6.

As established previously, genomic or posttranslational dysregulation of PDX1 is linked to cancer and diabetic phenotypes. In addition to the contribution of PDX1 to polygenic diabetes (T2D), PDX1 is also implicated in pancreatic agenesis, neonatal diabetes, and MODY phenotypes (143, 147, 148). In particular, several heritable mutations in *PDX1* exons have been identified by genetic testing of MODY4 and neonatal diabetes patients (Table 2 and Fig. 4*A*).

Phenotypic severity associated with *PDX1* mutation usually depends on the mutation type and zygosity. For instance, a frameshift mutation leading to PDX1 truncation N-terminal to the DBD is a causative factor in mild MODY4 (heterozygous) and neonatal diabetes resulting from pancreatic agenesis (homozygous) (9, 144, 149, 150, 151). Similar studies in MODY and neonatal diabetes patients revealed amino acid substitutions within the DBD that are predicted to impair PDX1 target gene activation in development and in mature β cells *via* decreased DNA binding (10, 135, 152, 153, 154, 155, 156, 157). In such cases, the onset of diabetes could arise from insufficient β cell mass and/or diminished glucose-driven activation of *INS* by PDX1 as a consequence of attenuated DNA binding.

Interestingly, the disease-linked mutations in PDX1 are not exclusive to the DBD, which further emphasizes the important roles (some yet unidentified) of the PDX1 IDRs (Fig. 4*A*). PDX1 harboring a C18R or P33T mutation in the N-terminal TAD leads to impaired β cell differentiation from pancreatic progenitor cells and decreased insulin, glucagon, and somatostatin activation in mature cells (56, 135). PDX1 mutations Q59L, D76N, and A140T were found in patients with T2D and may predispose the carrier to late onset T2D but are not believed to be causative (136, 158). A handful of other mutations in the PDX1 N-terminal IDR (and one in the C-terminal IDR) have been reported in diabetic patients but are similarly unlikely to be solely causative of diabetes (152, 159, 160) (Table 2).

Mutations in the TAD tend to be justifiably linked to impaired transcription activation, potentially from weakened interactions associated with or required for promoter accessibility or cooperative activation. Mutations that affect the PDX1 C-terminal IDR are more mechanistically elusive. Genetic testing in diabetic patients found G212R substitutions in patients with early onset, but not late onset, diabetes (MODY4). Another C-terminal substitution, P239Q, was identified in a patient with MODY3 and was linked to a more severe diabetic phenotype compared with the MODY3 mutation alone (145).

PDX1 with proline insertion at position 243 or point mutation resulting in E224K both had decreased transcription activation ability compared with the WT, but neither mutation was deemed causative (136, 161). Notably, E224K is the only C-terminal mutation that lies near to one of only two annotated protein-interaction motifs and appears to impair this interaction (118). Recurrence of specific mutations in the C-terminal IDR in different MODY patients, namely G212R, P239Q, and P243PP, raises the presumed likelihood of a causative association, yet begs the question of mechanism, given that these mutations do not overlap with either of the only two known motifs for binding within PDX1-C.

Table 2 contains the PDX1 clinical mutations documented to date and their corresponding references. Additional mechanistic studies toward linking clinically observed mutations to diabetic phenotypes will not only provide potential therapeutic targets but will also give insight into the many and changing roles of PDX1 in developing and mature organisms.

## PDX1 regulation *via* PTMs

As emphasized by the disease consequences of dysregulation of PDX1 or the genes under its control, appropriate regulation of PDX1 function is integral to organism health. Accordingly, intrinsically disordered transcription factors, such as PDX1, are subject to many layers of regulatory control.

So far, we have established that import of glucose by the β cell leads to (i) PDX1 association with the *INS* promoter, (ii) recruitment of chromatin remodelers and additional transcription factors, and ultimately, (iii) activation of *INS* expression. For the latter to occur, however, requirement is that PDX1 tune its intermolecular interactions for gene activation. While it is generally recognized that PDX1 transactivation potential is regulated through glucose-linked kinase pathways, the specific PTMs that direct PDX1 activation *versus* inhibition are not well established (11, 162, 163). The majority of the reported PDX1 PTMs are not linked to the interaction of a specific binding partner but instead to the effect of a given PTM on PDX1 transactivation potential. PTMs of proteins is an important mechanism toward justifying the vast and diverse protein functions that arise from a relatively small set of protein-coding genes (164). Table 3 summarizes the reported site-specific PTMs of PDX1.

**Table 3:** PDX1 residues reported to be modified to modulate PDX1 stability and/or transactivation potential

Location in PDX1	Modification	Modified residue	Predicted enzyme(s)	Reported effect(s)	Ref.
N terminus	Phosphorylation	T11	DNA-PK	Destabilizing	(175)
		S61	GSK3, ERK1/2, SAPK2	Destabilizing^a^, activating^b^	(176)^a^, (163)^b^
		S66	GSK3, ERK1/2, SAPK2	Destabilizing^a^, activating^b^	(176)^a^, (163)^b^
	Methylation	K123	Set7/9	Activating	(82)
		K131	Set7/9	Activating	(82)
DNA-binding domain	Phosphorylation	T151	PASK	Impairs nuclear import	(68)
C terminus	Phosphorylation	T213	HIPK2	Activating	(200)
		T230	CK2	Activating^c^, destabilizing^d^, stabilizing^e^	(182)^c^, (183)^d^, (71)^e^
		S231	CK2	Activating^c^, destabilizing^d^, stabilizing^e^	(182)^c^, (183)^d^, (71)^e^
		S268	HIPK2, GSK3	Localization to nuclear periphery^f^, destabilizing^g^	(69)^f^, (122, 123)^g^
		S272	GSK3	Destabilizing	(122)

The reported effects and predicted enzyme for each modification summarize findings from particular references indicated by a superscripted letter. ‘Destabilizing’ refers generally to shortened Pdx1 half-life and ‘stabilizing’ refers to increased PDX1 half-life. SUMOylation and O-GlcNacylation are reported (separately) to confer stabilizing and activating effects to PDX1; modified residues were not reported (186, 187).

Superscripts a through g link the associated reference where contradictory reported effects appear in the literature.

PTMs are integral to protein function for IDPs and folded proteins alike; however, compared to their folded counterparts, IDPs are generally overrepresented as targets of such modifications (165, 166, 167). Indeed, PTMs are integral to many of the same functions that leverage IDRs for regulation of the cell cycle, transcription, and signal transduction (48, 168, 169). Protein phosphorylation on Ser or Thr residues is one of the most common PTMs, especially in signaling-related processes, and notably, centers on two amino acids that are enriched and easily accessible in IDRs (48, 169). Phosphorylation within IDRs or IDPs is a versatile switch that allows reversible control over the interface presented to a potential binding partner. In many cases, phosphorylation events direct the presentation of a specific sequence or structural motif to interaction partners (170, 171, 172). Thus, PTMs in response to changing cellular conditions support IDPs in their numerous and diverse context-dependent roles (173, 174).

### Phosphorylation within the PDX1 TAD

Phosphorylation events in the PDX1 TAD are primarily implicated in the control of PDX1 transcription factor activity. However, evidence of upregulation or downregulation through specific phosphorylation events is contradictory in the literature. Additionally, although most PTMs in PDX1-N are activating in nature, phosphorylation of PDX1 threonine 11 does not directly affect transactivation potential but appears to mark PDX1 for proteasomal degradation in response to DNA damage (175). Alignment of the PDX1 sequences from different organisms may yield insights into the relative importance of the reported phosphorylation events and advance ongoing efforts to establish a model for PDX1 activity through its PTMs (Fig. 4*C*). Threonine 11 (T11) is highly conserved in mammals only, despite the modest conservation of the flanking amino acids in other species.

Serine residues 61 and 66 (S61 and S66) lie within the TAD and their phosphorylation by ERK1/2 in response to glucose stimulus enhances PDX1 transactivation (163). A separate study proposed that S61/S66 phosphorylation by a different kinase, glycogen synthase kinase 3 (GSK3), has no effect on transactivation ability but leads to PDX1 degradation (176). In support of the former, phosphorylation within the TAD of another pancreatic transcription factor, MafA, increases association with the coactivator β2; similarly, recall that p300 preferentially interacts with a TAD-phosphorylated form of PDX1 (88, 96).

In contrast to the studies implicating ERK1/2 or p38 Map kinase as modifiers of PDX1 function, overexpression of p38 or upstream kinases in this pathway had no apparent effect on PDX1 localization or transcription factor activity regardless of cellular glucose concentration. Instead, an activated form of PI3K was sufficient to drive PDX1 localization to the nucleus and transcriptional activation of *INS.* Given these results, it is possible that activation of PI3K by glucose may drive activation of downstream kinases in a pathway that is independent of p38/SAPK2 and ERK1/2 (177). In line with this model, the glucagon-like peptide (GLP-1) increases PI3K activity and that of adenylyl cyclase resulting in increased cellular cAMP concentrations and, subsequently, activation of PKA (178, 179). It is not known whether PKA directly phosphorylates PDX1 or if PKA activation indirectly contributes to PDX1 function; however, the sequence motif for many PKA targets, R-R-X-S, does not appear in the PDX1 sequence (180).

Multiple sequence alignment shows that S61 and S66 are conserved in the majority of organisms, except some species of fish (Fig. 4*C*). Notably, the residues surrounding these phosphorylation sites are not directly conserved but maintain remarkable chemical similarity across species. This pattern is consistent with a model for a careful balance of acidic and hydrophobic residues within TADs, in which conservation of amino acid identity is less important than maintaining the more general charge and hydrophobicity features of a TAD sequence (181).

Although we know that glucose-dependent phosphorylation events in the PDX1 TAD tailor recruitment of certain factors as a manner of *insulin* regulatory control, the signals that lead to these modifications, many of the enzymes responsible, and the specific mechanistic consequences remain unclear. Similarly, there have been no reports of the enzyme(s) responsible for the dephosphorylation of either PDX1 IDR.

### Phosphorylation in the PDX1 C-terminal IDR

The PDX1 C-terminal IDR contains four putative phosphorylation sites; the phosphorylatable residues all lie within motifs recognized by the only known PDX1-C-interacting protein, SPOP. However, different studies report different consequences for phosphorylation: threonine 230 (T230) and serine 231 (S231) were initially proposed to be targets of casein kinase 2 (CK2) phosphorylation toward PDX1 turnover and subsequent lowered transcriptional activity (182, 183). Studies of glucose-dependent phosphorylation of T230 and S231 posited that such modification should affect binding to SPOP and reported enhanced binding of SPOP to phosphomimetic mutants of PDX1 and decreased binding to SPOP upon inhibition of CK2 (183). In contrast, *in vitro* measurements of binding affinity to SPOP revealed that pT230 and pS231 modifications preclude SPOP binding and, thus, stabilize PDX1 (71). In either case, phosphorylatable residues at positions 230 and 231 are found across a range or organisms, suggesting an evolutionary pressure to retain this motif (Fig. 4*C*). In addition, conservation of these phosphorylation sites and the nearby residues is also likely important for regulation of PDX1 stability *via* the SPOP interaction motif that encompasses T230 and S231.

The remaining two sites of phosphorylation, S268 and S272, lie in another SPOP-binding (SB) motif. Sequence alignment shows high conservation of both sites in mammals but the absence altogether of this motif in some species of bird and fish (Fig. 4*C*). Although the proposed kinase differs between studies, phosphorylation of S268, S272, or S268/S272 appears to drive proteasomal degradation of PDX1 (122, 123). One such study evaluated the phosphorylation state of PDX1 in high and low glucose conditions and reported the low glucose–dependent phosphorylation of S268 and S272 by glycogen synthase kinase 3 (GSK3); of the two, S268 phosphorylation was reported to promote PDX1 degradation (122). This finding was later recapitulated in a phosphoproteomics study in which GSK3 activation inhibited PDX1 activity through phosphorylation of PDX1 S268 (123).

Interestingly, another study suggested the HD-interacting protein kinase 2 (HIPK2) as the enzymatic source of the low glucose phosphorylation of S268 but reported localization of phosphorylated PDX1 to the nuclear periphery as the repressive mechanism, which may yield degradation-independent suppression of PDX1 activity (69). The mechanisms by which PDX1-C phosphorylation—especially at S268/S272—affects PDX1 function are largely unknown. Future studies into the atomistic interactions between PDX1 and SPOP at this SB motif with and without phosphorylation may help discern the role of S268/S272 phosphorylation. Importantly, we should not exclude the possibility that there is a yet unidentified binding partner that may be sensitive to phosphorylation at one or both S268 and S272.

### Other PTMs of PDX1

Phosphorylation within the HD at position T151 is reported to control nuclear import of PDX1. In experiments using phosphomimetic (T151D) or phosphodeficient (T151A) mutants of PDX1, PDX1 harboring T151D was predominantly localized to the cytosol regardless of glucose stimulus. In this model, PDX1 phosphorylated by Per-Arnt-Sim kinase (PASK) at T151 (pT151) may be excluded or exported from the nucleus (68). Putative PTM events in PDX1-N and PDX1-C (and one within PDX1-HD) and their reported impacts are shown in Table 3 and Fig. 4. Alignment of PDX1 sequences from different organisms showed that T151 is highly conserved (Fig. 4*C*). Importantly, however, it is not clear whether this conservation is suggestive of a similarly conserved phosphorylation event or a consequence of high sequence homology across HD folds (184).

While serine and threonine phosphorylation events comprise the majority of the studied PTMs, other types of modifications are implicated in the regulation of PDX1 transcription factor activity. As discussed in the context of PDX1 protein–protein interactions, the methyltransferase Set7 is recruited to PDX1-N and modifies the histone H3 tail (80). Set7 has numerous nonhistone methylation substrates, such as the tumor suppressors p53 and RB (81, 185). Similarly, Set7 reportedly methylates PDX1 Lys123 and Lys131 in *in vitro* assays. Of the two, methylation of Lys131 conferred increased transcriptional activity in luciferase reporter assays; however, the mechanism by which this modification impacts PDX1 transactivation potential is not clear (82).

In line with PDX1 downregulation by proteasomal degradation, PDX1 is polyubiquitinated in cells but the lysine residue(s) that are modified to this end are yet unknown (72, 118). Given numerous lysine-associated modifications that are implicated in PDX1 function, the locations of lysines in PDX1 are indicated in Fig. 4*B*. PDX1 modification with the small ubiquitin-like modifier (SUMO) protein associated with a phosphorylation event was proposed, in part, based on anomalous SDS-PAGE migration of PDX1 isolated from β-like cells compared with PDX1 expressed in *Escherichia coli*. The lysine residue that is modified by SUMOylation was not reported, but knockdown of SUMO-1 reduced PDX1 transcriptional activity in reporter assays and decreased PDX1 half-life in cells (186). Another study of PDX1 isolated from β-like cells found that PDX1 was modified by *O*-linked *N*-acetylglucosamine (*O*-GlcNAc) and that this modification was associated with high-glucose conditions and insulin secretion. Modification of Ser/Thr by glycosylation may compete with phosphorylation events; however, the sites of modification by *O*-GlcNAc groups were not reported (187).

In order to establish rigorous connections between glucose conditions, PDX1 PTMs, and metabolic outcomes (*i.e*., insulin secretion), studies of PDX1 PTMs must not be limited to identification of modifications. Instead, efforts should focus on describing the enzymes responsible for installation and removal of modifications and the molecular level consequences of PTMs in the different domains of PDX1.

## Outlook

PDX1 is a master orchestrator of pancreatic transcription. In developing organisms, PDX1 controls the temporally precise differentiation of pancreatic β cells. In mature organisms, PDX1 maintains β cell identity and directs glucose-stimulated insulin secretion. Given the relevance of PDX1 to development and disease, PDX1 research efforts have been largely centered on clinical and organismal biology, but there remains much to be learned from molecular-scale studies of PDX1 function. Of general relevance for both physiological and disease-associated function is characterization of PDX1-containing complexes, assigning phenotype to clinically associated mutations, and decoding the functions of PTMs.

The structural properties of the PDX1 N- and C-terminal regulatory domains long posed major challenges to their mechanistic study; however, the recent emergence of biophysical techniques suited specifically for IDPs and IDRs should enable interrogation of the Pdx1 IDRs on the molecular level. Solution-state NMR spectroscopy remains the most tractable technique for the interrogation of IDRs with atomic resolution; however, technical limitations mean that the same strategies that are optimal for the study of IDRs are often incompatible with IDR-containing complexes at high resolution.

Importantly, we and others demonstrated that cocrystallization of fragments of the PDX1 C-terminal IDR with the substrate-binding domain of SPOP is a feasible approach to establishing an atomistic binding model (71, 72). Similar approaches may be leveraged toward specific complexes involving the N-terminal IDR following determination of suitable PDX1 peptide boundaries that support stable binding and crystallization. Technical and computational advances in the cryo-EM field now enable the study of smaller systems with higher resolution. Although cryo-EM has been used with success to solve structures of IDR-containing protein complexes, electron density for the highly flexible regions is still generally weak or absent (188, 189). However, it may be feasible to assemble a complex of PDX1, DNA, and purified coactivator proteins in which parts of the PDX1 IDRs might be stably bound. Although it is unlikely that this strategy would yield complete density for the IDRs, cryo-EM of such a complex could complement atomistic NMR studies and help to establish the spatial configuration of a PDX1 complex capable of gene activation.

Determining the specific binding modes of PDX1 and its various interacting partners may provide insight into the effects of specific diabetes-associated mutations and PTMs. It is also plausible that certain mutations or modifications may influence PDX1 interactions with factors that have not yet been identified. Further, assignment of PDX1 mutations observed in diabetic patients to mechanisms of PDX1 dysregulation should have an outsized impact on the therapeutic strategies in individuals with monogenic (MODY4) or T2D.

Of particular important focus toward understanding the signals that lead to PDX1 transcriptional activation is the study of the enzymes responsible for the placement and removal of PDX1 PTMs and the spatial and temporal specificity with which they do so. As a result, we may link glucose condition-specific PTM events with enhanced or impaired interactions with the modified regions of PDX1. By evaluating the existing literature surrounding PDX1 modifications and the PDX1 interactome, we may begin to make hypotheses about the possible effects of some PTMs and, thus, how PTMs exert control over PDX1 function.

Testing one such hypothesis may, for example, reveal a mechanism for the stabilizing effect of pRB binding to PDX1. The lysine residue(s) modified within the PDX1 ubiquitin-mediated degradative pathway is unknown; however, pRB binding to the PDX1 HD (see Fig. 2) reportedly protects PDX1 from polyubiquitination and proteasomal degradation (70). Accordingly, perhaps pRB association with PDX1 confers stability by physically occluding one or more ubiquitin-modifiable lysines (shown in pink in Fig. 4*B*). Separately, the proposed methylation events in the PDX1 N-terminal IDR could serve a similar role by blocking ubiquitination with an existing PTM.

The several PTMs, with emphasis on phosphorylation, are high-value targets of study toward understanding the glucose-linked signals that lead to PDX1 upregulation or downregulation. However, as evidenced by contradictory enzyme assignments and ambiguous consequences on PDX1 functions, we must explore *in vitro* approaches to complement *in vivo* studies of PDX1 phosphorylation. NMR is a tractable strategy to study possible structural consequences of PDX1 PTMs; we and others have leveraged NMR strategies toward kinetic and structural characterization of PTMs in disordered protein systems (190, 191, 192, 193).Through such approaches, we may generate new hypotheses for PTM-regulated PDX1 function and, further, parse the findings from within very complex cellular systems.

Decades of work toward clinical descriptions of PDX1 function enables us now to connect healthy and disease phenotypes to specific molecular mechanisms of transcriptional control. In addition, study of the PDX1 IDRs in this context may be generally applicable to other IDR-containing transcription factors and shed new light on the many layers of regulation of proteins involved in gene expression processes.

## Conflicts of interest

The authors declare that they have no conflicts of interest with the contents of this article.
